# Artificial intelligence framework for multi-pathology risk assessment from retinal fundus images: deep learning approach to 15-disease screening

**DOI:** 10.3389/fmed.2026.1778404

**Published:** 2026-05-25

**Authors:** Robert Vasilev, Andrey Savchenko, Pavel Blinov, Tadej Svetina, Stepan Kudin, Nikolay Romanenko, Yuliya Sarana, Gleb Khizhnyak, Andrey Demchinsky, Taisia Shcheglova

**Affiliations:** 1 Z-union AI Technologies Consortium, Moscow, Russia; 2 Department of the Problems of Physics and Power Engineering, Moscow Institute of Physics and Technology, Dolgoprudny, Russia; 3 Sber AI Lab, Moscow, Russia; 4 HSE University, Laboratory for Theoretical Foundations of AI Models, Moscow, Russia; 5 Skolkovo Institute of Science and Technology, Center for Bio- and Medical Technologies, Moscow, Russia; 6 Association for Specialists in Progress, Engineering, and Clinical Technologies Unified by Medicine 'ASPECTUM', ELVIS Neuroimplants LLC, Moscow, Russia

**Keywords:** class imbalance, computer-aided diagnosis, deep learning, disease risk assessment, fundus photography, medical image analysis, retinal imaging

## Abstract

**Background:**

Automated disease screening systems face challenges when applied to multi-class medical image analysis, particularly under severe class imbalance inherent in clinical datasets. Retinal fundus imaging enables non-invasive screening for multiple ocular and systemic diseases simultaneously, yet current automated systems typically assess risk for only a single pathology or a limited disease range. We developed a comprehensive AI framework for simultaneous risk stratification of 15 distinct pathological conditions from retinal fundus images and report its preliminary evaluation in a real-world clinical setting.

**Methods:**

Our system combines fundus images from publicly available sources and proprietary clinical archives, addressing the significant class imbalance challenge inherent in rare condition risk assessment. We employed a hybrid deep learning architecture (CAFormer B36) with focal loss and targeted oversampling to ensure robust performance across common and rare conditions. The framework identifies retinal biomarkers associated with both primary ocular diseases (diabetic retinopathy, glaucoma, macular degeneration, cataracts, retinitis pigmentosa) and systemic conditions with retinal manifestations (hypertensive retinopathy, atherosclerotic changes, autoimmune manifestations). Dataset splitting was performed at the image level; all internal metrics should therefore be interpreted as exploratory upper-bound estimates pending patient-level replication.

**Results:**

On the internal image-level test set, the system achieved ROC AUC of 0.9524–0.9971 across all 15 pathological classes, with F1 scores of 0.8968–0.9649. Notably, rare conditions with fewer than 100 training examples demonstrated robust risk stratification performance. In an exploratory single-site evaluation on 68 real-world cases, overall accuracy was 64.7% (95% CI: 52.9–76.5%). Due to the very limited number of sight-threatening cases in this small cohort (glaucoma *n* = 2, diabetic retinopathy *n* = 4), sensitivity estimates for these categories carry extremely wide confidence intervals and cannot be considered statistically reliable. These findings underscore the need for larger, prospective, multicenter studies.

**Conclusions:**

This pilot proof-of-concept demonstrates the feasibility of multi-pathology risk stratification under severe class imbalance using a hybrid deep learning architecture. Internal image-level metrics show strong discriminative capability, while the preliminary single-site evaluation (*n* = 68, 64.7% accuracy) reveals the challenges of real-world translation and the substantial gap that remains before clinical deployment. This work provides a methodological foundation for future prospectively validated screening systems.

## 1 Introduction

Non-communicable diseases account for nearly 74% of global mortality, with vision-threatening and systemic conditions representing a major preventable burden (1). Approximately 2.2 billion people worldwide live with vision impairment, and at least 1 billion of these cases are preventable through early detection and timely intervention (2). The leading causes of preventable blindness—diabetic retinopathy, glaucoma, age-related macular degeneration, and cataracts (3–6)—share a critical feature: their prognosis depends heavily on how early they are identified. Alongside these primary ocular diseases, retinal imaging also captures biomarkers of systemic conditions with microvascular manifestations, including hypertensive retinopathy, atherosclerotic vascular changes, and autoimmune retinopathies, making fundus photography a uniquely information-rich non-invasive window into both ocular and systemic health.

The retinal vasculature and neural tissue provide a unique opportunity for non-invasive assessment, as the retina is the only site where blood vessels and neural tissue can be directly visualized without surgery (7). Pathological changes in retinal microvasculature, optic nerve structure, and macular architecture serve as biomarkers for cardiovascular disease, neurodegenerative disorders, and autoimmune conditions. This capability enables opportunistic screening for multiple disease risks during routine ophthalmic examinations, a prospect with significant implications for primary care and population health.

Recent advances in artificial intelligence and deep learning have enabled remarkable progress in automated risk assessment through retinal fundus imaging (8). Landmark studies have demonstrated high predictive accuracy for diabetic retinopathy, glaucoma, and age-related macular degeneration (9). Contemporary research has expanded toward foundation models capable of detecting multiple retinal conditions simultaneously (10, 11), with transformer-based architectures showing particular promise for multi-label classification tasks (12). However, automated detection of systemic disease risks manifesting through retinal changes remains an active area of investigation (13–15), with significant potential for early screening of cardiovascular, neurological, and autoimmune conditions.

Despite these advances, most current platforms are limited to narrow disease ranges due to model architecture constraints and training limitations. Existing systems typically focus on single pathologies or small subsets of related conditions, limiting their clinical utility in comprehensive screening scenarios (16). Furthermore, the challenge of severe class imbalance in retinal disease datasets, where rare conditions are significantly underrepresented, remains inadequately addressed in current literature. Most published studies rely on publicly available datasets that, while valuable, do not fully represent the diversity of pathological presentations encountered in real clinical practice.

To address these limitations, we developed a comprehensive, automated, multi-disease risk stratification platform capable of distinguishing among 15 distinct pathological conditions, encompassing both primary ocular diseases and systemic conditions with retinal manifestations. Our approach is distinguished by several key innovations. First, we assembled a unique dataset combining publicly available fundus images with proprietary clinical data from partner healthcare institutions, including rare pathological conditions that are typically underrepresented in research datasets. Second, we developed specialized annotation protocols in collaboration with board-certified ophthalmologists, focusing on pathognomonic features and subtle biomarkers indicative of elevated disease risk. Third, we implemented advanced training strategies specifically designed to handle severe class imbalance while maintaining high sensitivity for rare but clinically significant conditions.

A distinctive aspect of our study is the construction of a dataset that deliberately includes rare pathologies, such as retinoblastoma, systemic lupus manifestations, and atherosclerotic vascular changes, which are critically underrepresented in existing public benchmarks. By capturing these edge-case conditions alongside common retinal diseases, the dataset provides a realistic testbed for assessing model robustness and clinically relevant failure modes in multi-disease screening scenarios.

Our approach leverages the CAFormer B36 architecture (17), a hybrid model that combines convolutional neural network capabilities with transformer-based attention mechanisms. Through systematic comparative analysis, we demonstrate superior performance compared to established architectures for both global and local feature extraction, which are crucial for retinal pathology identification.

The main contributions of this work are as follows. First, we developed training techniques that address severe class imbalance (up to 95:1 ratio), enabling the model to learn from as few as 49–89 training examples per rare class. Second, we demonstrate that the CAFormer B36 hybrid architecture outperforms ResNet50 (18), EfficientNet-B3 (19), ViT-B16 (20), and Swin Transformer (21) on this multi-label task, which we attribute to its ability to combine local convolutional feature extraction with transformer-based global context. Third, we report a preliminary single-site evaluation on 68 real-world cases (64.7% overall accuracy), providing an initial indication of behavior outside the training distribution while explicitly acknowledging its statistical limitations. Fourth, we commit to publicly releasing an annotated dataset subset and the source code to support reproducibility. Taken together, this work is a pilot, proof-of-concept study: its purpose is to demonstrate feasibility and map out the key design decisions that future large-scale validation studies will need to address.

It is important to emphasize that our system performs risk stratification and screening rather than clinical diagnosis. The detection of retinal biomarkers associated with various pathological conditions indicates elevated risk requiring further clinical evaluation, not definitive disease confirmation. This distinction aligns with appropriate clinical workflows where automated screening tools support but do not replace comprehensive medical assessment by qualified healthcare professionals.

## 2 Materials and methods

### 2.1 Dataset construction and annotation

We assembled a comprehensive dataset of 20,351 retinal fundus images using a multi-source approach that combined publicly available repositories (12,000 images, 59%) with proprietary clinical data (8,351 images, 41%). This hybrid strategy enabled the capture of rare pathological conditions typically underrepresented in standard research datasets.

#### 2.1.1 Data sources

Public datasets (12,000 images): ODIR-5K (4,200 images), RFMiD Dataset (2,800 images), APTOS 2019 (3,200 images), Kaggle DR Detection (1,100 images), and supplementary sources including MESSIDOR, STARE, and DRIVE (700 images).

Proprietary clinical data (8,351 images): Images collected through institutional collaboration agreements between 2018–2024:

- Center for Laser Medicine network (Yoshkar-Ola, Cheboksary, Russia): Primary proprietary source providing rare systemic manifestations, including hypertensive retinopathy (*n* = 49 training), systemic lupus manifestations (*n* = 66 training), atherosclerotic changes (*n* = 59 training), plus common pathologies. Imaging: Topcon TRC-50DX and Zeiss Visucam systems.
- Private ophthalmology clinics (Moscow): Multiple tertiary centers contributing across all major pathology classes. Imaging: Heidelberg Spectralis and Canon CR-2 systems.

All proprietary data were collected in accordance with institutional review board protocols, with retrospective analysis of anonymized imaging data.

#### 2.1.2 Data splitting and critical limitation

Dataset division employed stratified random sampling, maintaining class proportions: training 70% (seed = 42), validation 15% (seed = 43), test 15% (seed = 44).

Patient-level grouping procedure: To mitigate the data leakage risk inherent in image-level splitting, we developed a two-stage pseudo-patient grouping procedure applied prior to dataset partitioning.

In the first stage, near-duplicate images were identified using perceptual hashing (pHash). A 64-bit hash was computed for each image; pairs with a Hamming distance of 8 bits or fewer were considered duplicates and merged into a single group.

In the second stage, images from the same patient, acquired across different sessions, were identified by exploiting the uniqueness and temporal stability of the retinal vascular pattern. For each image, a vessel map was computed using the Frangi vesselness filter. Keypoints were extracted from the vessel maps using the ORB (Oriented FAST and Rotated BRIEF) descriptor; matches between pairs of vessel maps were refined using RANSAC. Pairs of images with at least 25 geometrically consistent inliers (RANSAC matches) were classified as belonging to the same patient.

All identified pairs were merged into groups using a Union-Find (Disjoint Set Union) structure. The resulting pseudo-patient groups were used as stratification units during dataset partitioning: the training, validation, and test sets were constructed using GroupShuffleSplit, ensuring that all images within the same pseudo-patient group were assigned exclusively to one partition.

Residual limitation: The pseudo-patient grouping procedure relies on vascular pattern similarity and therefore cannot guarantee perfect patient-level separation in all cases. Images with poor vessel visibility (e.g., dense cataracts), very similar pathological patterns across different patients, or insufficient overlap between sessions may result in ungrouped singletons or missed matches.

Patient-level test set results: To quantify the impact of the pseudo-patient grouping procedure, we evaluated three independently trained model variants on the resulting patient-safe test set (*n* = 3,981 images; GroupShuffleSplit partitioning). Results are reported in Supplementary Table S1. Under this stricter partitioning, mean AUC decreased from 0.976 (image-level) to 0.934 (range across three variants: 0.930–0.939), confirming that a portion of the image-level performance reflects patient-level memorization. The three model variants showed high inter-run consistency (AUC range ≤ 0.008, macro-F1 range ≤ 0.008), demonstrating training stability across different experimental configurations. Classes with strong discriminative signals (Glaucoma: AUC 0.995, Macular degeneration: 0.982, Retinitis Pigmentosa: 0.986) retained high AUC under patient-level conditions. Classes representing rare systemic conditions (Atherosclerosis, Hypertensive retinopathy, Systemic lupus) showed substantially lower F1 scores (0.32–0.42), consistent with the small training sample sizes and the association-based nature of their ground truth labels.

Implication for reported metrics: All image-level results in the main text should be interpreted as upper-bound estimates. The patient-level metrics in Supplementary Table S1 provide a more conservative and methodologically rigorous characterization of model performance.

#### 2.1.3 Annotation protocol

Images annotated by board-certified ophthalmologists specializing in medical retina, glaucoma, and uveitis. Each image is reviewed by ≥2 independent experts, and discrepancies are resolved through consensus. Inter-annotator agreement (*n*= 200 validation subset): primary ocular diseases κ = 0.89, rare conditions κ = 0.81, systemic manifestations κ = 0.76.

#### 2.1.4 Class definitions

To ensure reproducibility, we define ambiguous nomenclature:

CHPRE – *Central High-risk Peripheral Retinal Evaluation*: Lattice degeneration, retinal breaks, atrophic holes, vitreoretinal traction posing rhegmatogenous detachment risk.

Malformation – Congenital abnormalities: optic disc coloboma, chorioretinal coloboma, morning glory anomaly, retinal dysplasia, persistent fetal vasculature.

Pigmented Choroidal Neoplasm – Melanocytic lesions from benign nevi to uveal melanomas, graded by thickness (>2mm), subretinal fluid, orange pigment, and documented growth.

Atherosclerotic vascular changes – Retinal biomarkers: generalized arteriolar narrowing (AV ratio < 0.6), focal narrowing, AV nicking (Scheie grade ≥2), arteriolosclerotic changes (copper/silver wiring).

Systemic lupus manifestations – Retinal findings in documented SLE: cotton-wool spots, hemorrhages, vascular sheathing, microvasculopathy, serous detachments.

Hypertensive retinopathy – Keith-Wagener-Barker grade ≥2: AV nicking, focal narrowing, hemorrhages, exudates, cotton-wool spots.

#### 2.1.5 Ground truth for systemic disease categories

Atherosclerotic changes (*n* = 95): Images from patients with documented cardiovascular disease (coronary artery disease, peripheral arterial disease, cerebrovascular events confirmed by medical records). Annotations identified retinal vascular biomarkers only. Validation: cardiologist review (30 cases, 90% agreement). Inter-rater κ = 0.82.

Hypertensive retinopathy (*n* = 84): Confirmed hypertensive patients (BP ≥140/90 mmHg on ≥3 measurements within 6 months of imaging). Retinal signs graded Keith-Wagener-Barker ≥2. Inter-rater κ = 0.87.

Systemic lupus manifestations (*n* = 110): Rheumatologist-confirmed SLE (ACR/EULAR criteria, positive ANA and anti-dsDNA). Retinal lupus-associated findings annotated. Inter-rater κ = 0.79.

Critical caveat: These annotations represent *association* between retinal biomarkers and documented systemic conditions in known-positive populations. We did *not* validate predictive accuracy for detecting undiagnosed systemic disease in screening populations. Our model identifies retinal risk indicators warranting systemic evaluation, not definitive systemic diagnosis.

#### 2.1.6 Dataset composition

Table 1 presents the final distribution across training, validation, and test sets. The dataset exhibits substantial imbalance reflecting clinical prevalence: well-represented classes (Normal: 4,274, Peripheral Retinal Degeneration: 3,955, Macular Degeneration: 3,218) vs. rare conditions (atherosclerotic changes: 95, hypertensive retinopathy: 84, retinoblastoma: 89). The imbalance ratio is approximately 95:1, necessitating specialized training strategies.

**Table 1:** Composition of the fundus image database.

Class/ pathology	Training	Validation	Test
Normal	2,560	857	857
Atherosclerotic vascular changes	59	15	21
CHPRE	450	155	156
Cataract	977	321	337
Diabetic retinopathy	330	114	100
Glaucoma	1,753	580	584
HIV-related retinopathy	143	46	52
Hypertensive retinopathy	49	17	18
Macular degeneration	1,920	644	654
Malformation	60	21	21
Peripheral retinal degeneration	2,377	783	795
Pigmented choroidal neoplasm	911	314	319
Retinitis pigmentosa	311	102	106
Retinoblastoma	52	19	18
Systemic lupus manifestations	66	21	23
Vascular occlusions	158	57	48

The dataset combines publicly available images with proprietary clinical data from partner institutions, particularly for rare conditions.

#### 2.1.7 Data availability

Following publication, we commit to public release: (1) annotated subset (1,200 images, minimum 50 per class where feasible), (2) preprocessing code, (3) annotation protocols, (4) metadata. Data hosted on Zenodo with a persistent DOI under a CC-BY 4.0 license.

*Limitation*: Proprietary clinical images (41% of dataset) cannot be released due to institutional agreements. The released subset will prioritize rare pathologies underrepresented in existing public datasets.

### 2.2 Data pre-processing pipeline

We developed a systematic preprocessing pipeline to standardize input images while preserving clinically relevant features. Initial analysis revealed models could exploit acquisition artifacts rather than retinal pathology, necessitating careful preprocessing design.

#### 2.2.1 Multi-label classification framework

Critical clarification: This work addresses retinal disease assessment as multi-label classification, not multi-class. Each image may present risk indicators for multiple coexisting pathologies (e.g., diabetic retinopathy with hypertensive changes).

Formally, each image **x**_*i*_ is associated with a 15-dimensional binary vector $y_{i}\in {\left\{0,1\right\}}^{15}$, where *y*_*i, j*_ = 1 indicates presence of risk indicators for pathology *j*. Images without detectable pathologies are encoded as **y**_*i*_ = **0** (referred to as “Normal” for clinical interpretability).

The model produces 15 independent sigmoid outputs ${\hat{y}}_{i}\in {\left[0,1\right]}^{15}$ (not softmax), trained with binary cross-entropy loss per dimension. During inference, an image is classified as Normal when all 15 probabilities fall below τ = 0.5.

Important: In results tables, “Normal” appears as a separate class for clinical interpretability, but this does not represent a 16th class—it is simply the condition where all pathology scores are below threshold.

For metric computation, each of the 15 pathological categories is treated as an independent binary label within the multi-label framework. The “Normal” category is defined operationally as the subset of images for which all 15 ground truth labels are zero, i.e., no pathological risk indicators are present. Metrics for “Normal” thus quantify the model's ability to correctly identify images without any active pathological labels, rather than performance on a separate mutually exclusive class in a softmax sense.

#### 2.2.2 Preprocessing pipeline

The standardized pipeline consists of four stages:

1. Image standardization: Aspect-ratio-preserving resize to 224 pixels (shorter dimension), followed by center crop to 224 × 224. This resolution balances computational efficiency with preservation of retinal details.

*Acknowledged limitation*: 224 × 224 resolution may discard fine-grained features (e.g., early drusen, small microaneurysms) visible in full-resolution images, representing a trade-off between computational tractability and diagnostic precision.

2. Background elimination: Circular mask applied to eliminate peripheral artifacts, camera borders, institutional logos, and device-specific characteristics. Pixels outside the fundus field-of-view are set to zero.

3. Data augmentation (training only, online random application):

- Geometric: horizontal flip (*p* = 0.5), rotation [−15°, +15°], translation/shear up to 10%
- Photometric: brightness/contrast × [0.8, 1.2], saturation × [0.85, 1.15], Gaussian blur (*p* = 0.2)

Augmentation applied *after* oversampling, ensuring diversity despite class balancing replication.

4. Normalization: ImageNet statistics [μ=[0.485,0.456,0.406], σ = [0.229, 0.224, 0.225]].

### 2.3 Model architecture design

**Figure 1:**
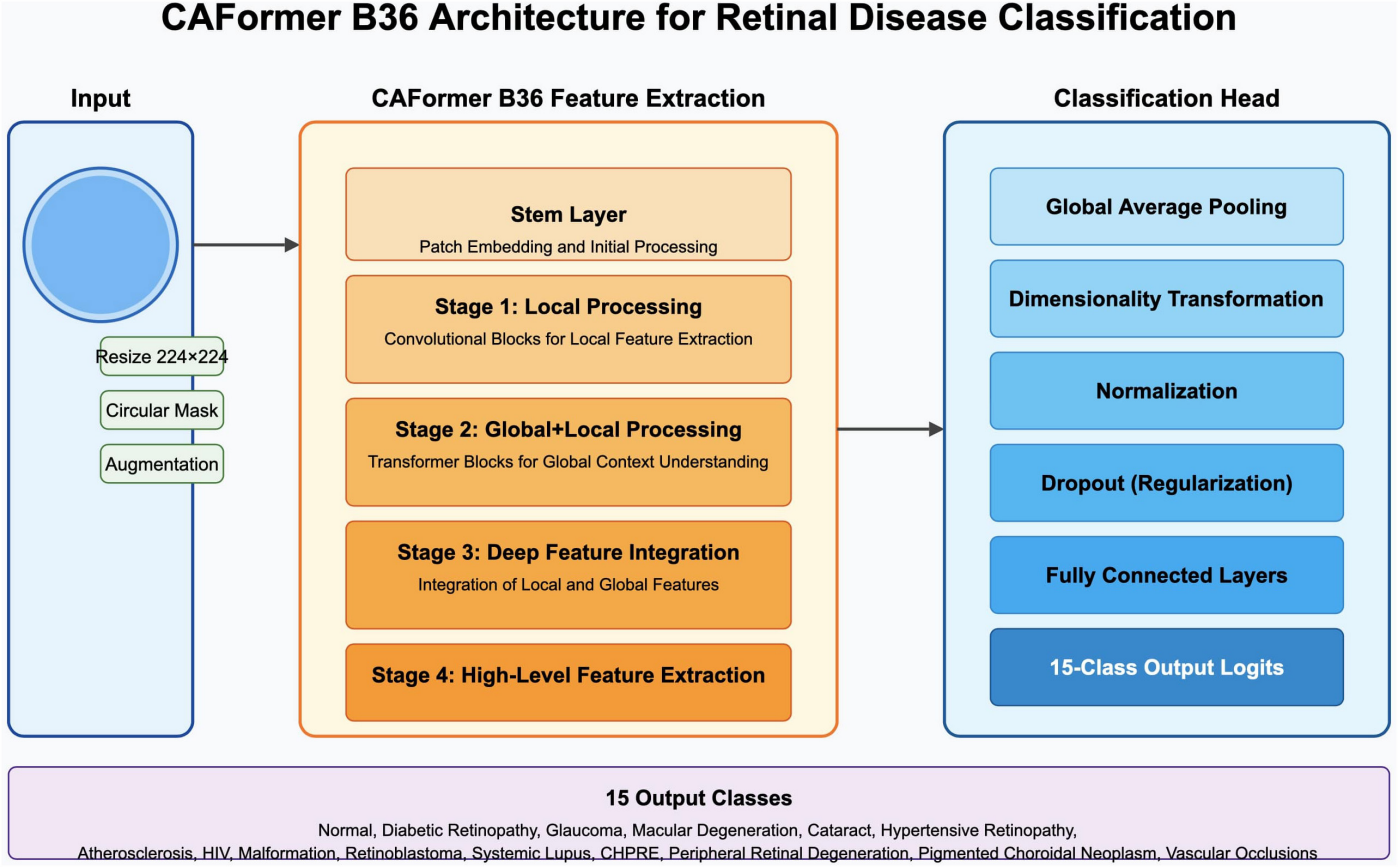
Pipeline architecture for multiclass disease prediction from retinal images using CAFormer B36.

We selected the CAFormer B36 architecture (17) as our primary model (Figure 1) due to its demonstrated effectiveness in combining the strengths of convolutional neural networks with transformer-based global attention mechanisms. This hybrid approach proves particularly valuable for medical imaging applications requiring both fine-grained local feature detection and comprehensive global context understanding.

The CAFormer B36 model leverages transfer learning from large-scale pretraining on ImageNet-22K, followed by fine-tuning on ImageNet-1K, providing robust visual feature representations that transfer effectively across domains between natural and medical images.

To validate our architectural choice, we conducted systematic comparisons with established baselines: ResNet50 (18), EfficientNet-B3 (19), Vision Transformer ViT-B16 (20), and Swin Transformer (21). The comparative analysis confirmed CAFormer B36 superiority in balancing local feature extraction capabilities with global context modeling.

### 2.4 Training strategy and optimization

The training methodology addresses severe class imbalance (a 95:1 ratio in the rarest classes) through strategic data resampling, specialized loss functions, and advanced optimization techniques.

#### 2.4.1 Class imbalance mitigation

Random oversampling: We employed RandomOverSampler from the imbalanced-learn library to balance class representation during training. Important clarification: RandomOverSampler *does* duplicate minority class examples at the dataset level—this is its intended mechanism. However, diversity is maintained through *strong online data augmentation* applied after oversampling. Each duplicated example undergoes random geometric and photometric transformations during training, ensuring the model rarely encounters identical image presentations across epochs. Here are the concrete oversampling ratios applied for various classes: Rare classes (< 100 examples): 5 × oversampling; Medium classes (100–500 examples): 3 × oversampling; Common classes (>500 examples): no oversampling.

Class-weighted augmentation: Augmentation intensity scaled inversely with class size—rare conditions received more aggressive augmentation compared to common conditions.

#### 2.4.2 Specialized loss functions

We employed a composite loss function combining two components:

1. Focal Loss: Dynamically adjusts loss contribution based on prediction confidence, emphasizing hard-to-classify examples from underrepresented classes. Focusing parameter γ = 2.0 down-weights well-classified examples, while balancing factor α = 0.25 adjusts for class imbalance.


$$\begin{array}{l} L_{\text{focal}}\left(p_{j}\right)=-\alpha_{j}{\left(1-p_{j}\right)}^{\gamma }\log\left(p_{j}\right) \end{array}$$ (1)

2. Partial Binary Cross-Entropy: Standard BCE (Binary Cross-Entropy) loss filtered to exclude weak negative signals (predicted probability < 0.05).

The total loss is a weighted sum of the focal and BCE components: $L_{\text{total}}=0.7\cdot L_{\text{focal}}+0.3\cdot L_{\text{partial-BCE}}$

#### 2.4.3 Optimization configuration

Optimizer: Adam with β_1_ = 0.9, β_2_ = 0.999, weight decay 10^−4^ for L2 regularization.

Learning rate schedule: CosineAnnealingLR with initial learning rate ${\text{lr}}_{\text{init}}=10^{-4}$, minimum learning rate ${\text{lr}}_{\text{min}}=10^{-6}$, providing smooth decay over 30 epochs.

Training configuration: 30 epochs, batch size 32. The best checkpoint was selected by peak validation mAP (achieved at epoch 29, mAP = 0.8998; macro-F1 = 0.8806). Training dynamics are shown in Supplementary Figure S3.

Hardware: NVIDIA A100 GPUs with Automatic Mixed Precision for memory efficiency.

Training time: Approximately 6 h for 30-epoch training.

#### 2.4.4 Critical training limitations

No k-fold cross-validation: Due to computational constraints and dataset size, we employed a single train/validation/test split rather than k-fold cross-validation. This introduces uncertainty in performance estimates, particularly for rare classes where statistical power is limited.

Single training run: Results reported from the best-performing checkpoint of a single training run (not averaged across multiple runs with different seeds). This may introduce optimistic bias.

Validation on same-device images: Validation and test sets contain images from the same acquisition devices and institutions as the training set (though different patients). True cross-device and cross-institutional generalization requires prospective external validation.

#### 2.4.5 Training dynamics and convergence

Training exhibited stable convergence: loss dropped sharply in the first 5 epochs and plateaued below 0.001 by epoch 10. Validation mAP improved steadily throughout, reaching its peak of 0.8998 at epoch 29. Rare classes showed slower improvement relative to common classes, consistent with the expected difficulty of learning from small positive samples, yet continued to improve through the final epochs without signs of overfitting (train and validation loss remained closely aligned throughout; see Supplementary Figure S3).

## 3 Results

### 3.1 Model performance analysis

The comparative evaluation demonstrated the superior performance of CAFormer B36 in multi-class retinal disease risk stratification. Our systematic analysis across 15 distinct pathological conditions revealed exceptional predictive capabilities, with the model achieving ROC AUC scores ranging from 0.9524 to 0.9971 across all disease categories.

Table 2 presents comprehensive performance metrics for each disease class, illustrating remarkable consistency in predictive performance. Particularly noteworthy is exceptional performance on rare conditions. Retinoblastoma, represented by only 89 total images, achieved an impressive ROC AUC of 0.9878 with an F1-score of 0.9336. Similarly, hypertensive retinopathy with only 84 examples demonstrated robust performance metrics, with an ROC AUC of 0.9617 and an F1-score of 0.9433. Precision–recall curves for all 15 classes are provided in Supplementary Figure S4. ROC curves per class are shown in Supplementary Figure S2 (mean AUC = 0.976). A summary of per-class average precision, AUC-ROC, and F1 scores at optimal threshold is presented in Supplementary Figure S1 (mAP = 0.976, mAUC = 0.976, mF1 = 0.923).

**Table 2:** Risk stratification performance metrics for the CAFormer B36 model on the image-level test set across all 15 pathological categories.

Class	ROC AUC (95% CI)	F1 (95% CI)	Precision	Recall
Normal	0.9681 (0.958–0.978)	0.9360 (0.924–0.948)	0.9325	0.9330
Diabetic retinopathy	0.9854 (0.977–0.993)	0.9208 (0.906–0.935)	0.9181	0.9437
Glaucoma	0.9793 (0.971–0.987)	0.9117 (0.896–0.927)	0.9240	0.9254
Macular degeneration	0.9914 (0.985–0.997)	0.9565 (0.946–0.967)	0.9435	0.9426
Hypertensive retinopathy	0.9617 (0.942–0.981)^*^	0.9433 (0.916–0.971)^*^	0.9381	0.9207
Cataract	0.9971 (0.995–0.999)	0.9649 (0.956–0.974)	0.9735	0.9384
Retinitis pigmentosa	0.9718 (0.960–0.984)	0.9404 (0.926–0.955)	0.9536	0.9391
Atherosclerotic vascular changes	0.9683 (0.942–0.995)^*^	0.9042 (0.866–0.943)^*^	0.9057	0.9272
HIV-related retinopathy	0.9769 (0.964–0.990)	0.9358 (0.919–0.952)	0.9288	0.9318
Malformation	0.9846 (0.965–1.000)^*^	0.9308 (0.896–0.966)^*^	0.9307	0.9345
Retinoblastoma	0.9878 (0.967–1.000)^*^	0.9336 (0.895–0.972)^*^	0.9717	0.9196
Systemic lupus manifestations	0.9885 (0.970–1.000)^*^	0.9359 (0.904–0.968)^*^	0.9202	0.9525
CHPRE	0.9678 (0.957–0.979)	0.8968 (0.880–0.913)	0.9070	0.9145
Peripheral retinal degeneration	0.9609 (0.950–0.972)	0.9308 (0.919–0.943)	0.9342	0.9033
Pigmented choroidal neoplasm	0.9524 (0.940–0.965)	0.9189 (0.903–0.935)	0.9274	0.9248
Average	0.9761	0.9307	0.9339	0.9297

^*^Wide CI reflects small test-set support (*n* < 25); interpret with caution.

All metrics are reported as point estimates; 95% bootstrap confidence intervals (10,000 resamples) are provided in parentheses for ROC AUC and F1. These results are based on image-level splitting and should be interpreted as exploratory upper-bound estimates (see Subsection 2.1.2).

It is important to note that, for these rare classes, both training and test sets contain relatively few examples, and the primary split is performed at the image level. These factors increase the risk of optimistic bias in the reported metrics. Consequently, the metrics for rare categories should be interpreted as upper-bound estimates under the current experimental design, pending confirmation in larger, strictly patient-level and externally validated cohorts.

### 3.2 Feature visualization and interpretability analysis

**Figure 2:**
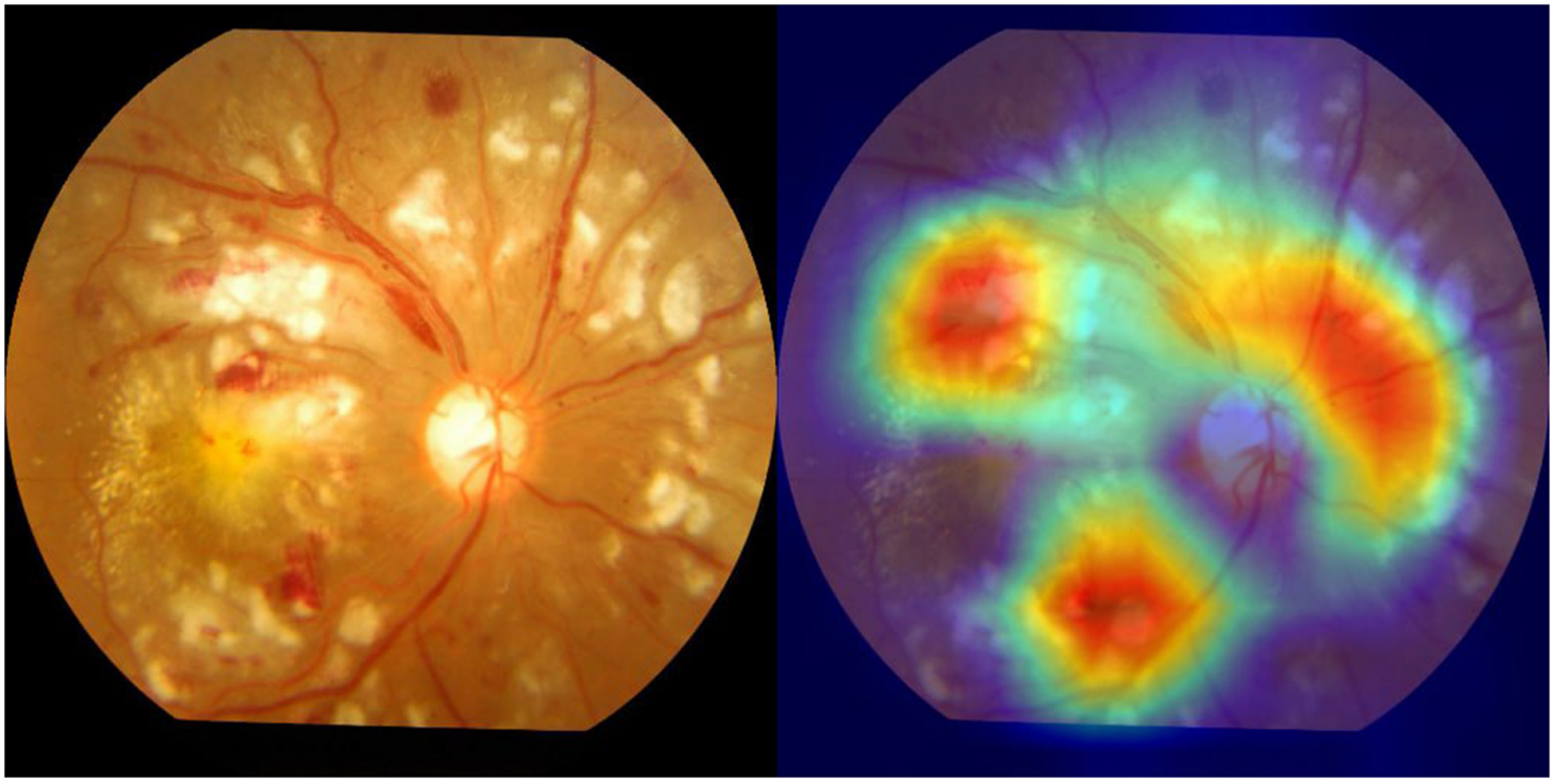
Model visualization for hypertensive retinopathy classification. The activation map highlights retinal hemorrhages, cotton-wool spots, and hard exudates, all characteristic of this systemic condition.

**Figure 3:**
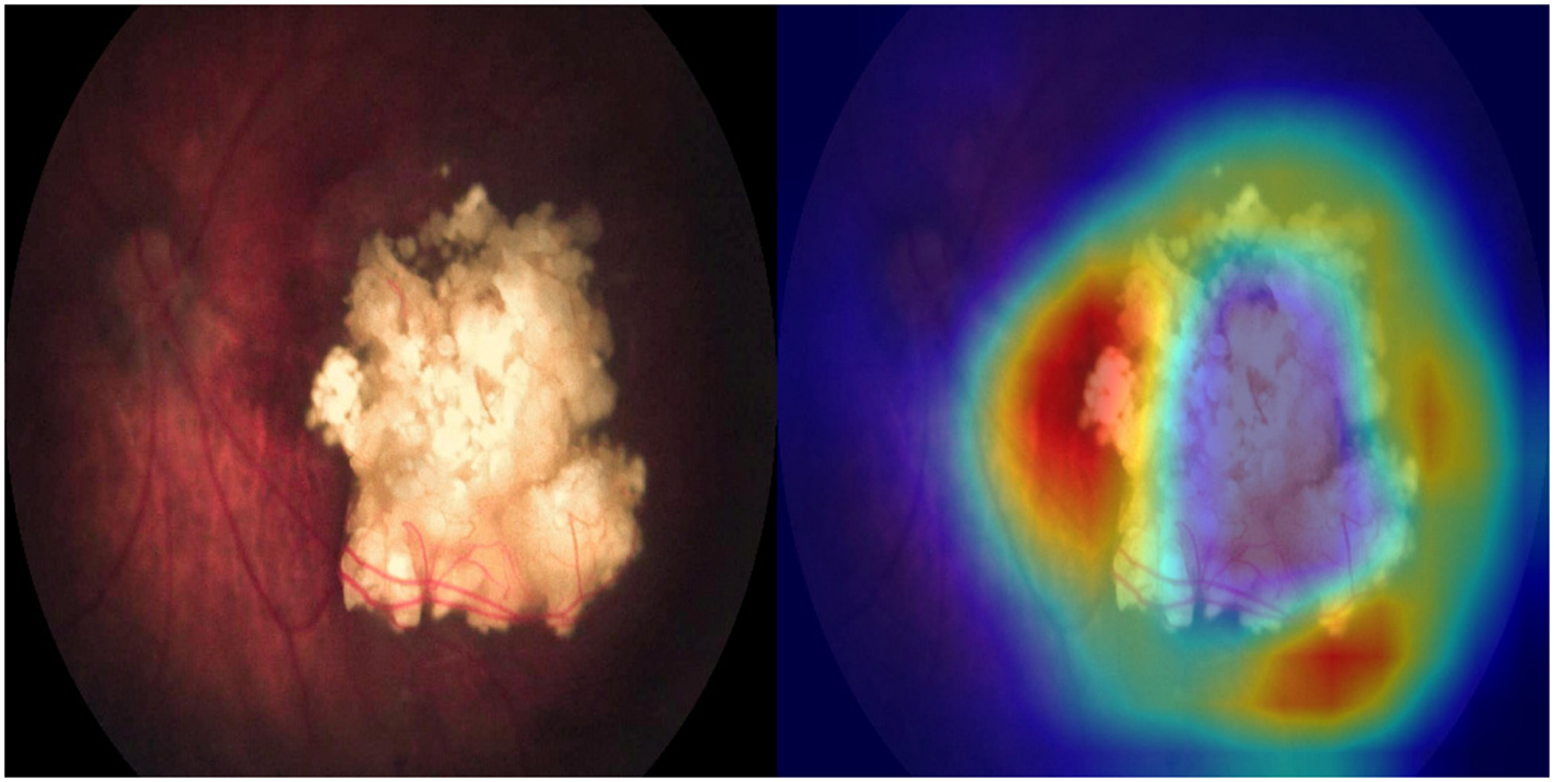
Retinoblastoma classification with feature localization. The model accurately identifies the focal volumetric formation with irregular borders characteristic of intraocular malignancy.

**Figure 4:**
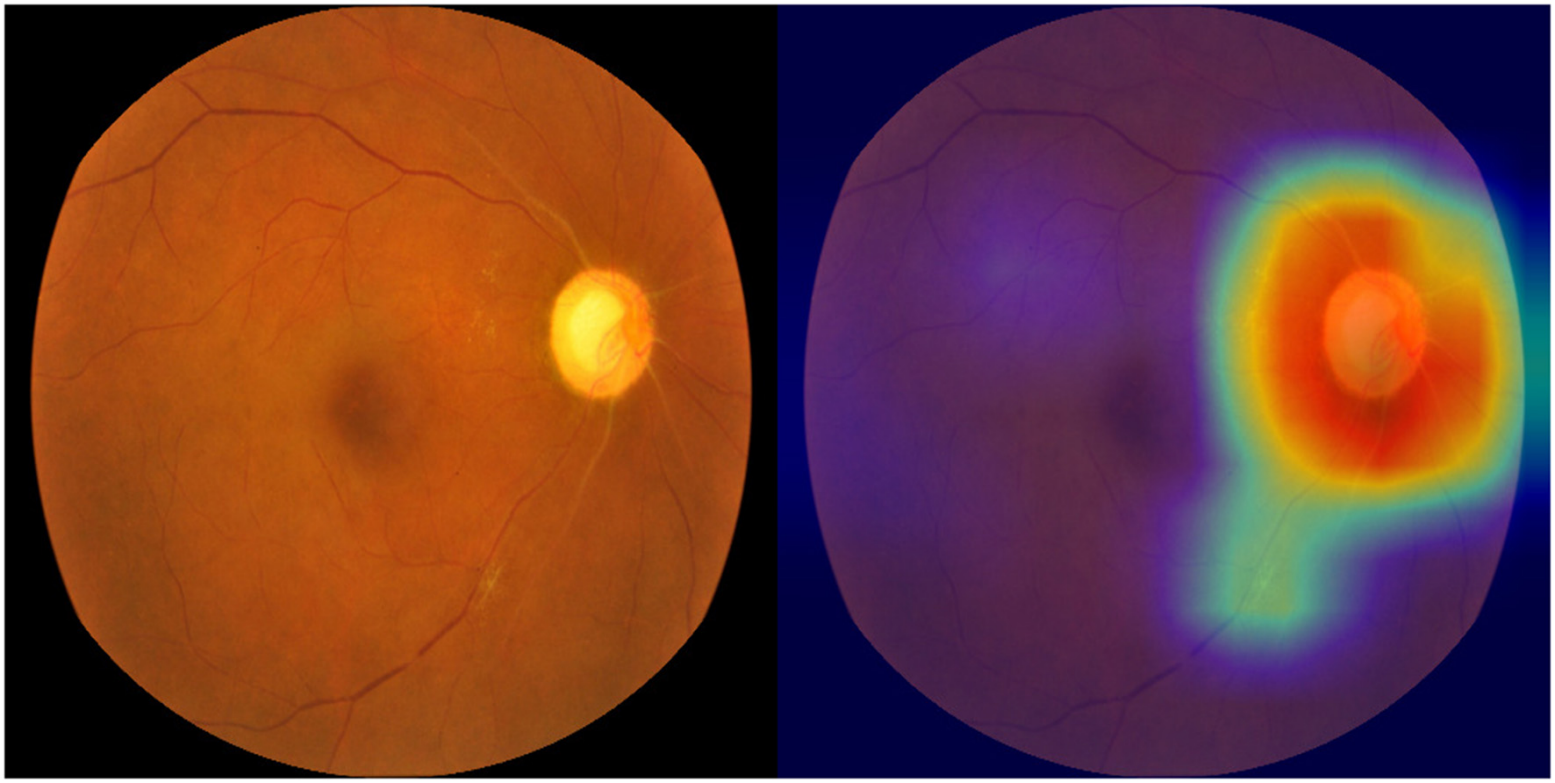
Glaucoma feature visualization showing increased optic disc excavation, neuroretinal rim thinning, and peripapillary changes consistent with glaucomatous optic neuropathy.

To enhance clinical interpretability, we employed gradient-weighted class activation mapping to visualize the anatomical regions that contribute most to risk stratification decisions. These visualizations confirm that CAFormer B36 focuses on clinically relevant pathological features rather than spurious correlations or acquisition artifacts. For hypertensive retinopathy risk assessment (Figure 2), the model accurately identified characteristic retinal biomarkers, including hemorrhages, cotton wool spots, and hard exudates. For retinoblastoma detection (Figure 3), the model successfully identified a characteristic focal mass with irregular contours. The glaucoma risk assessment (Figure 4) showcases the model's ability to detect increased optic disc cupping, neuroretinal rim thinning, and peripapillary atrophy.

### 3.3 Clinical validation study

Our exploratory clinical evaluation on a small convenience sample of 68 cases provides an initial indication of how the system behaves in a real-world setting, but should be interpreted as preliminary only. The validation dataset comprised: normal cases (35 images), retinitis pigmentosa (18 images), macular degeneration (5 images), diabetic retinopathy (4 images), cataract (3 images), glaucoma (2 images), and HIV-related retinopathy (1 image).

Clinical validation achieved an overall accuracy of 64.7%. The system demonstrated 100 percent sensitivity for sight-threatening conditions, including glaucoma and diabetic retinopathy, though this metric reflects only 6 positive cases (2 glaucoma + 4 diabetic retinopathy) with a wide confidence interval (95% CI: 54–100%), limiting statistical interpretability.

Table 3 presents detailed performance metrics across disease categories. All per-class metrics in Table 3 carry wide confidence intervals due to small support. Here are the key intervals (exact Clopper–Pearson method):

- Glaucoma sensitivity (100%, *n* = 2): 95% CI 15.8–100%. Clinically uninformative.
- Diabetic retinopathy sensitivity (100%, *n* = 4): 95% CI 39.8–100%.
- Sight-threatening conditions combined (100%, *n* = 6): 95% CI 54.1–100%.
- Overall accuracy (64.7%, *n* = 68): 95% CI 52.9–76.5%.
- Single-site design: all validation cases originated from the same institution as training data, using the same acquisition devices.

**Table 3:** Clinical validation results from an exploratory single-site evaluation (*n* = 68).

Disease class	Support	Precision	Recall	F1-score
Retinitis pigmentosa	18	100.0%	66.7%	80.0%
Glaucoma	2	66.7%	100.0%	80.0%
Diabetic retinopathy	4	36.4%	100.0%	53.3%
Macular degeneration	5	66.7%	80.0%	72.7%
Normal	35	71.4%	57.1%	63.5%
Overall accuracy	68	64.7%		

Support indicates the number of true-positive cases per class. All estimates carry wide confidence intervals due to small per-class sample sizes.

The causes of the gap between internal and clinical performance are analyzed in Subsection 4.2.

### 3.4 Comparative architecture analysis

Superior performance of CAFormer B36 compared to alternative architectures validates our architectural selection. Table 4 presents averaged performance metrics for five different architectures evaluated under identical training and evaluation protocols. Statistical analysis confirmed that performance differences between CAFormer B36 and alternative architectures were statistically significant with a p-value less than 0.05 across all major evaluation metrics, as assessed by paired t-tests on per-class performance measures.

**Table 4:** Comparative performance of different model architectures for retinal disease risk stratification, demonstrating CAFormer B36 superiority across all evaluation metrics.

Architecture	ROC AUC	F1	Precision	Recall
CAFormer B36	0.9761	0.9307	0.9339	0.9297
ResNet50	0.9329	0.8735	0.8802	0.8691
EfficientNet-B3	0.9265	0.8651	0.8873	0.8462
ViT-B16	0.9483	0.8962	0.9114	0.8845
Swin transformer	0.9412	0.8897	0.9065	0.8752

## 4 Discussion

### 4.1 Methodological contributions

The CAFormer B36 architecture outperformed all four baselines (ResNet50, EfficientNet-B3, ViT-B16, Swin Transformer) on this multi-label task, confirming that its hybrid design—local convolutional feature extraction combined with global transformer-based context—is a reasonable fit for retinal pathology detection, where both fine-grained local lesions and global structural patterns carry diagnostic weight. Recent work has also explored Swin Transformer variants for fundus disease detection (22) and GAN-based synthetic data augmentation to improve generalization under limited data conditions (23); the present study complements these directions by addressing the multi-label, multi-pathology setting under severe class imbalance.

The class imbalance mitigation strategy (focal loss + targeted oversampling + class-weighted augmentation) proved sufficient to train the model on classes with as few as 49 training examples while maintaining AUC above 0.96. This is methodologically relevant beyond the specific task: it demonstrates that hybrid oversampling with strong online augmentation can substitute for larger rare-class datasets in pilot settings, though the reliability of these rare-class estimates remains to be confirmed under patient-level and cross-site conditions.

A key emphasis of this work is the distinction between *risk stratification* and clinical diagnosis. The system outputs a probability vector across 15 pathological categories, identifying retinal biomarkers associated with elevated risk, not issuing diagnostic conclusions. This framing aligns with appropriate clinical workflows where automated tools triage and prioritize rather than replace specialist evaluation.

### 4.2 Internal-clinical performance gap: causes and implications

The gap between internal image-level test metrics (ROC AUC 0.95–0.99) and the preliminary single-site clinical evaluation (64.7% overall accuracy) is the most practically important finding in this paper, and warrants structured analysis rather than a single disclaimer.

We identify four contributing factors, listed in approximate order of expected impact:

(1) Patient-level data leakage. Image-level splitting allows multiple images from the same patient to appear in both training and test sets. The patient-level experiment (Supplementary Table S1, *n* = 3,981, GroupShuffleSplit) directly quantifies this effect: mean AUC decreased from 0.976 to 0.934 (Δ = 0.042), mAP from 0.976 to 0.642 (Δ = 0.334), and macro-F1 from 0.931 to 0.623 (Δ = 0.308). This confirms that a substantial portion of the image-level F1 and mAP reflects patient-specific memorization rather than generalization to unseen patients. AUC is more robust to this effect because it is threshold-independent, whereas F1 and mAP are sensitive to both memorization and the distribution shift introduced by patient-level rebalancing.

(2) Prevalence and distribution shift. The internal test set has a normal-case prevalence of approximately 21% (857/4,056 images), whereas the clinical validation cohort was 51% normal (35/68 cases). This mismatch shifts the operating point of a fixed-threshold classifier: a model calibrated for a 21% normal prevalence will over-predict pathology in a 51% normal population, directly degrading accuracy. A back-of-the-envelope calculation suggests that this alone could account for 8–12 percentage points of the observed accuracy gap.

(3) Domain shift and device heterogeneity. The clinical validation cohort was acquired in a real outpatient setting with naturally occurring image quality variation, patient cooperation variability, and potential use of devices not represented in the training set. Our preprocessing pipeline (circular masking, photometric normalization) partially mitigates device-specific artifacts, but was not systematically validated across the full range of fundus camera types encountered in clinical practice. Cross-device generalization is therefore an unaddressed risk.

(4) Shortcut learning. The exceptionally high AUC for cataract (0.9971) is likely driven in part by image quality degradation that accompanies cataract rather than retinal pathology *per se*. Analogous shortcuts may exist for other classes—for example, image-acquisition metadata embedded in public dataset images that correlate with disease labels. GradCAM visualizations (Subsection 3.2) show plausible anatomical focus for most classes, but GradCAM is not sufficient to rule out shortcut learning.

These factors are not mutually exclusive and are likely to combine. The practical implication is that the present internal metrics should be treated as an optimistic upper bound, not a deployment benchmark. Closing this gap will require, at minimum: (i) strict patient-level splitting across all data sources, (ii) prospective validation on an independent multi-site cohort with controlled prevalence, and (iii) cross-device evaluation with explicitly held-out device types.

### 4.3 Systemic disease assessment and dataset contribution

A notable aspect of the framework is its coverage of systemic conditions with retinal manifestations—hypertensive retinopathy, atherosclerotic vascular changes, and systemic lupus manifestations. These categories are largely absent from standard public fundus datasets, which is why we assembled a proprietary clinical subset specifically for them. The model's ability to detect retinal biomarkers associated with these conditions (AUC 0.962–0.989 on the internal test set) supports the concept of opportunistic systemic risk flagging during routine retinal screening, though this interpretation requires prospective validation in undiagnosed screening populations where ground truth cannot rely on pre-existing clinical records.

The dataset itself is a contribution. By deliberately including rare pathologies—retinoblastoma (*n* = 89), systemic lupus manifestations (*n* = 110), atherosclerotic changes (*n* = 95), hypertensive retinopathy (*n* = 84)—alongside the standard disease categories, it provides a more realistic testbed for multi-label classifiers than single-disease or few-disease public benchmarks. The planned Zenodo release (minimum 50 images per class from publicly releasable sources) will support comparison and reproducibility.

### 4.4 Limitations and future directions

The principal limitations are: (1) image-level splitting across all datasets, introducing optimistic bias of uncertain magnitude; (2) clinical validation cohort of only 68 cases from a single institution with inadequate statistical power for per-class conclusions; (3) no cross-device or cross-institutional validation; (4) binary classification without severity grading; (5) single training run without cross-validation; (6) no direct empirical comparison with retinal foundation models such as RETFound or EyeCLIP, which is a recognized gap that upcoming studies will address.

This study should be read as a pilot investigation—its contribution is to demonstrate that multi-pathology risk stratification under severe class imbalance is tractable and to map the design choices and failure modes that future, statistically powered studies will need to address. The essential next steps are patient-level prospective validation with hundreds of cases per major disease class, cross-device evaluation, integration of severity grading, and systematic comparison of foundation models.

## 5 Conclusions

This paper describes a pilot study on multi-pathology risk stratification from retinal fundus images, covering 15 ocular and systemic conditions under severe class imbalance. The CAFormer B36 architecture, trained with focal loss and targeted oversampling, achieved ROC AUC of 0.9524–0.9971 on the internal image-level test set and outperformed four established baseline architectures. These internal metrics are exploratory upper-bound estimates due to image-level (rather than patient-level) data splitting.

The preliminary single-site clinical evaluation (*n* = 68, 64.7% overall accuracy) revealed a substantial gap relative to internal test performance, which we attribute primarily to patient-level data leakage, prevalence mismatch, and domain shift. This gap is the central unsolved problem for the next stage of this work.

The dataset is a concrete contribution: it combines 12,000 public images with 8,351 proprietary clinical images, deliberately including rare categories (retinoblastoma, systemic lupus manifestations, atherosclerotic vascular changes) that are underrepresented in existing benchmarks. The planned Zenodo release will provide a resource for the community.

The system performs risk stratification, identifying retinal biomarkers of elevated pathological risk (not clinical diagnosis). For now, it is not ready for clinical deployment. The value of this work is methodological: it demonstrates that multi-label classification across 15 retinal conditions is feasible under severe class imbalance and maps the specific validation gaps that future prospective multi-site studies must address.

## Data Availability

The original contributions presented in the study are publicly available. This data can be found here: https://huggingface.co/andromoon17/z-retina. It includes representative samples from all 15 pathological categories, with a minimum of 20 images per category where available; expert annotations; preprocessing code and specifications; detailed documentation of annotation protocols; and metadata specifications. Additionally, source code for the model architecture, training scripts, evaluation protocols, and preprocessing pipelines is publicly available at https://github.com/sb-ai-lab/z_retina.

## Supplementary material

Supplementary Figure S1 Per-class performance summary on the image-level test set. Bar charts showing average precision (AP), AUC-ROC, and F1 score at optimal threshold for each of the 15 pathological categories. Dashed horizontal lines indicate macro-averaged values (mAP = 0.976, mAUC = 0.976, mF1 = 0.923). Pigmented Choroidal Neoplasm shows the lowest AP (0.949) and F1 (0.881), consistent with its relatively small training set and high inter-class visual similarity.

Supplementary Figure S2 ROC curves for all 15 pathological classes. Receiver operating characteristic curves computed on the image-level test set. Mean AUC = 0.976 (range 0.950–0.998). The dashed diagonal represents the random classifier baseline. Per-class AUC values are shown in the legend. All metrics are image-level upper-bound estimates (see Subsection 2.1.2).

Supplementary Figure S3 Training dynamics over 30 epochs. Left: ASL training and validation loss. Center: validation mean average precision (mAP). Right: validation macro-F1 at optimal per-class threshold. Best checkpoint selected at epoch 29 (mAP = 0.8998, macro-F1 = 0.8806). Train and validation loss curves remain closely aligned throughout, indicating no overfitting.

Supplementary Figure S4 Precision–Recall curves for all 15 pathological classes. Precision–recall curves computed on the image-level test set. Mean average precision (mAP) = 0.976 (range 0.949–0.998). High precision is maintained across a broad recall range for most classes, with a steeper drop-off for classes with fewer positive examples (Atherosclerosis, Hypertensive retinopathy, Peripheral Retinal Degeneration).

Supplementary Table S1 Performance on the patient-level test set (*n* = 3,981; GroupShuffleSplit partitioning). Three independently trained model variants were evaluated; mean and range (min–max) reported across variants. ^†^Classes with fewer than 100 positive test examples. For comparison, image-level metrics from Table 2: mean AUC = 0.976, mAP = 0.976, macro-F1 = 0.931.
