# Supplementary figures and images for: Artificial intelligence framework for multi-pathology risk assessment from retinal fundus images: deep learning approach to 15-disease screening
Source: Front Med (Lausanne). 2026 May 25;13:1778404. doi: 10.3389/fmed.2026.1778404 (PMC13244632; doi:10.3389/fmed.2026.1778404)

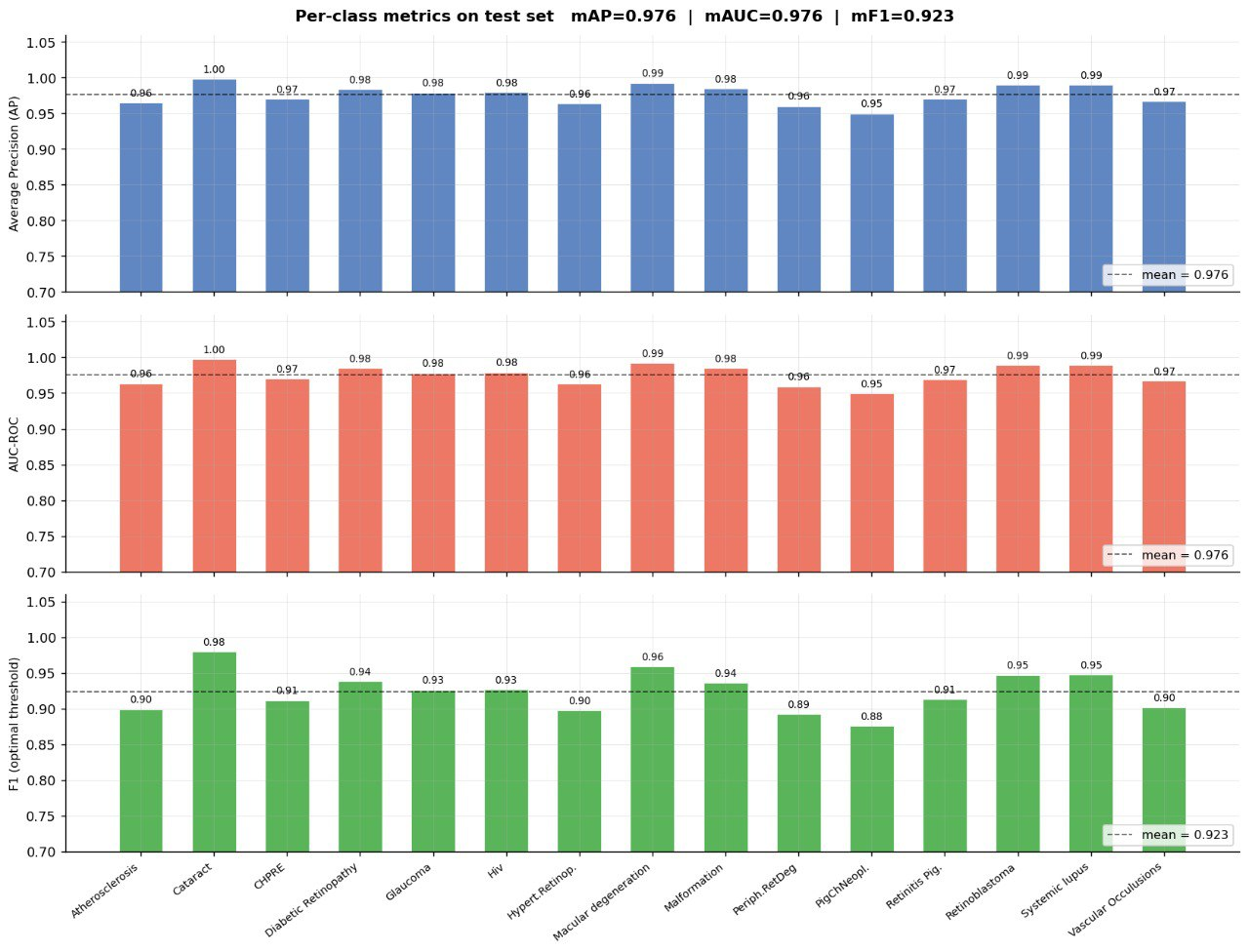

Supplement: Supplementary Figure S1 — Per-class performance summary on the image-level test set. Bar charts showing average precision (AP), AUC-ROC, and F1 score at optimal threshold for each of the 15 pathological categories. Dashed horizontal lines indicate macro-averaged values (mAP = 0.976, mAUC = 0.976, mF1 = 0.923). Pigmented Choroidal Neoplasm shows the lowest AP (0.949) and F1 (0.881), consistent with its relatively small training set and high inter-class visual similarity. [file Image_1.png]

## Supplementary Figure S2 — Training Dynamics

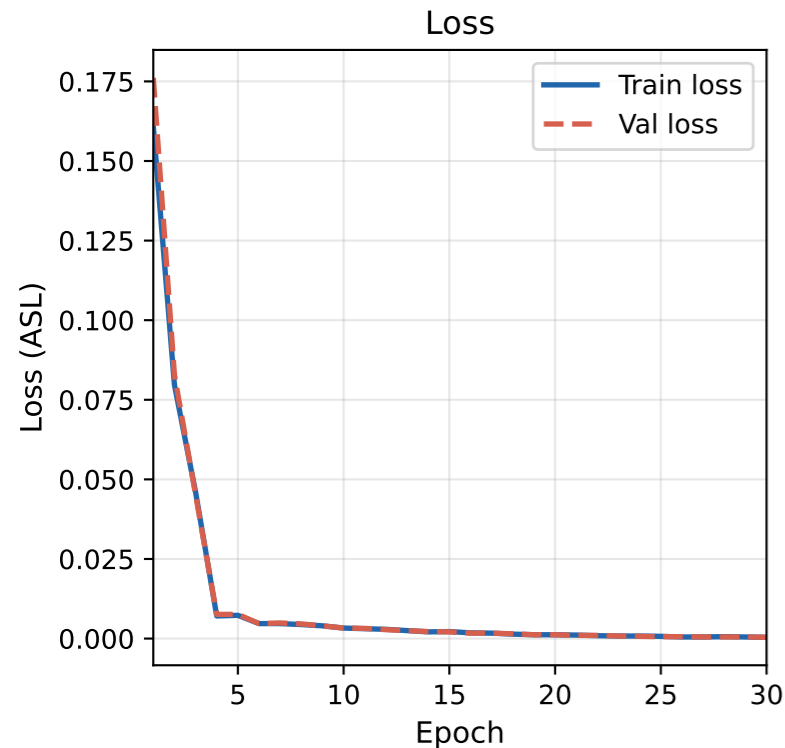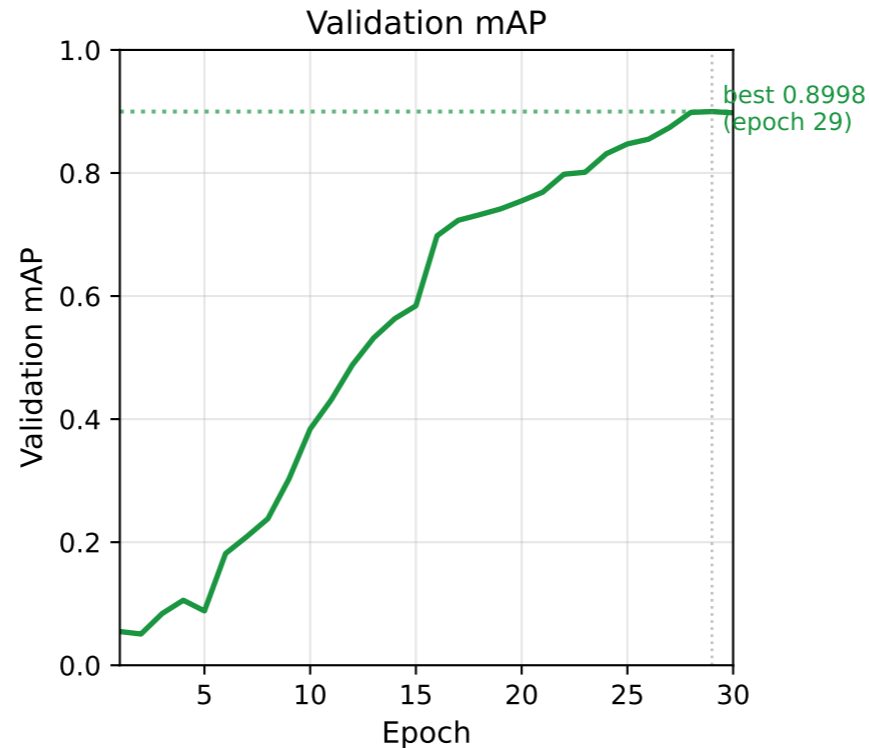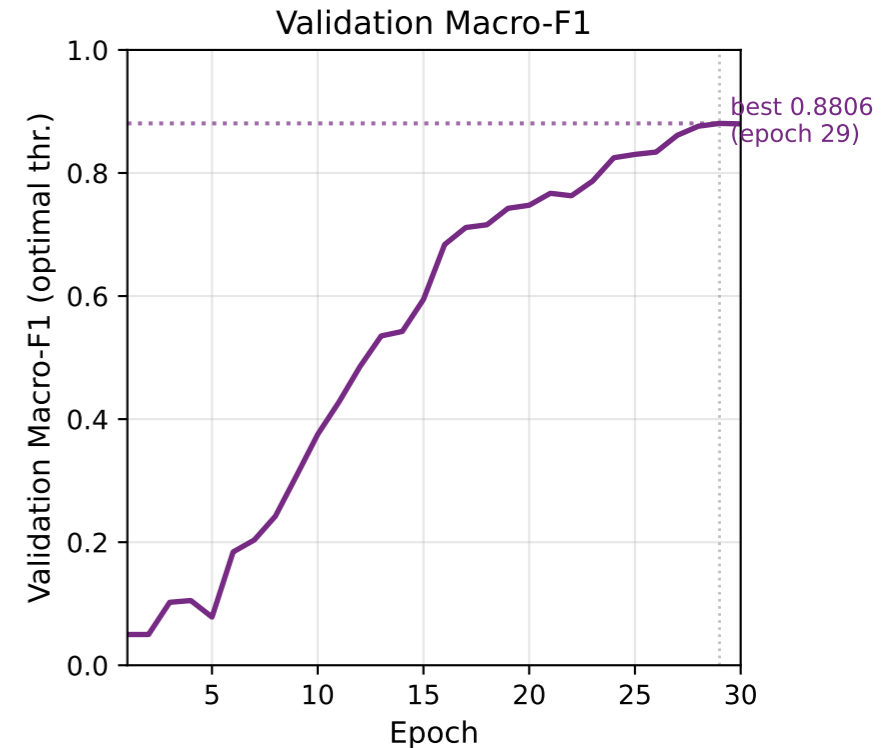

Supplement: Supplementary Figure S2 — ROC curves for all 15 pathological classes. Receiver operating characteristic curves computed on the image-level test set. Mean AUC = 0.976 (range 0.950–0.998). The dashed diagonal represents the random classifier baseline. Per-class AUC values are shown in the legend. All metrics are image-level upper-bound estimates (see Subsection 2.1.2). [file Image_2.pdf]

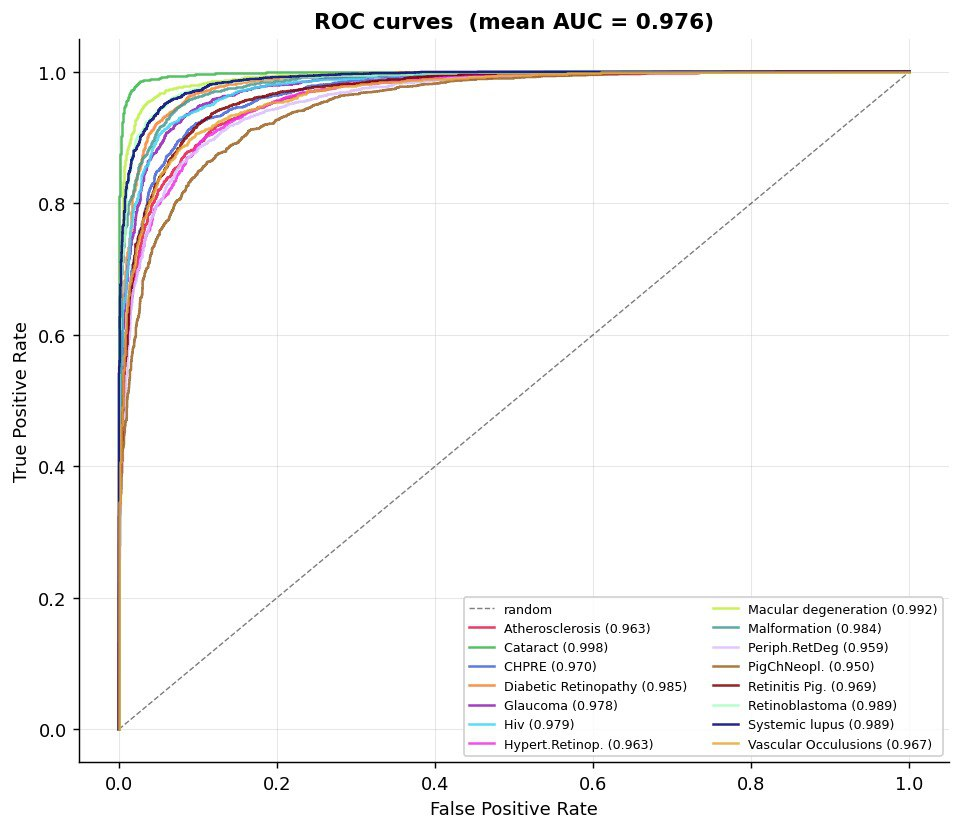

Supplement: Supplementary Figure S3 — Training dynamics over 30 epochs. Left: ASL training and validation loss. Center: validation mean average precision (mAP). Right: validation macro-F1 at optimal per-class threshold. Best checkpoint selected at epoch 29 (mAP = 0.8998, macro-F1 = 0.8806). Train and validation loss curves remain closely aligned throughout, indicating no overfitting. [file Image_3.png]

**Supplementary Figure S4 — Precision-Recall Curves**  
**mAP = 0.976**

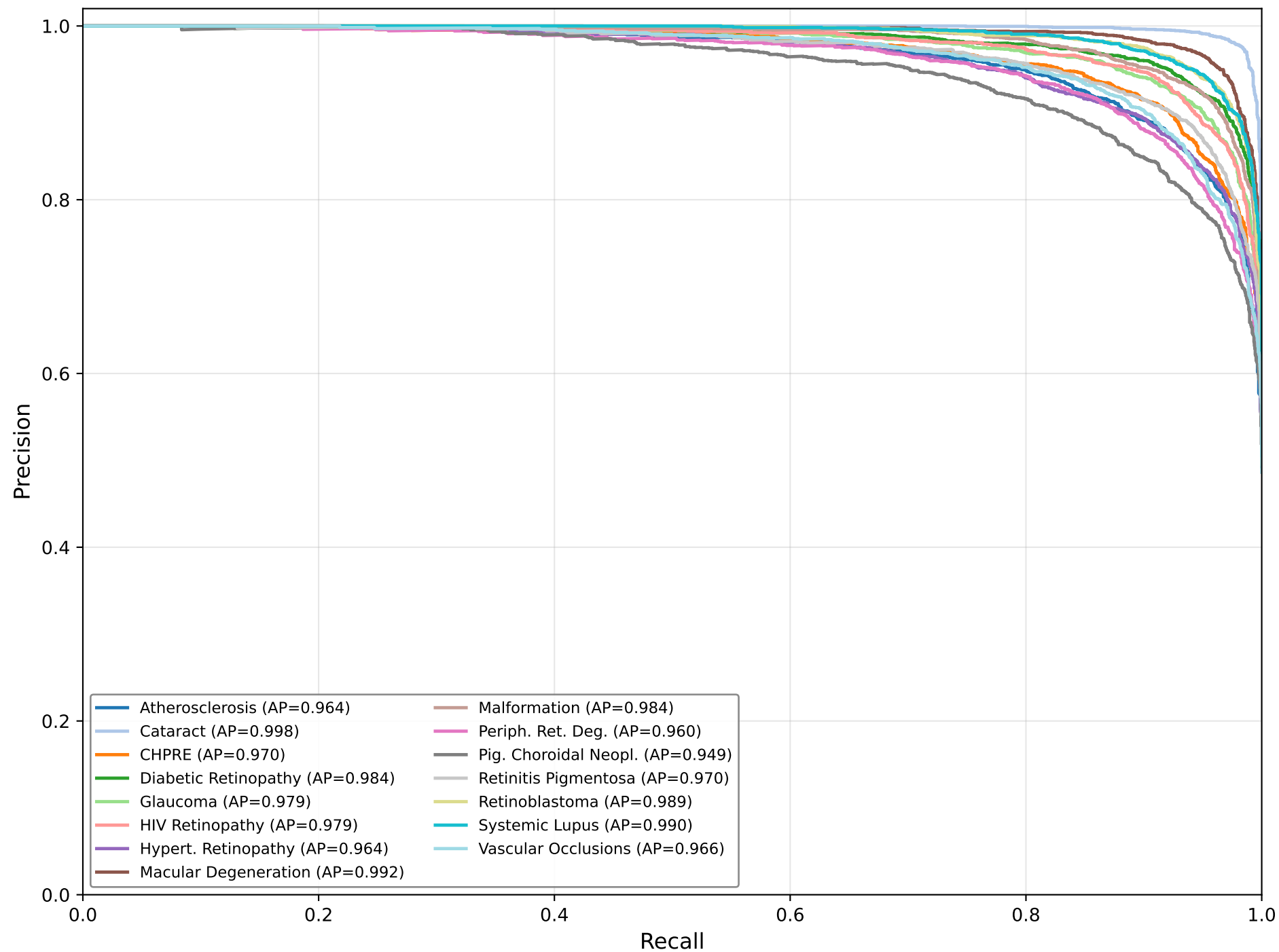

Supplement: Supplementary Figure S4 — Precision–Recall curves for all 15 pathological classes. Precision–recall curves computed on the image-level test set. Mean average precision (mAP) = 0.976 (range 0.949–0.998). High precision is maintained across a broad recall range for most classes, with a steeper drop-off for classes with fewer positive examples (Atherosclerosis, Hypertensive retinopathy, Peripheral Retinal Degeneration). [file Image_4.pdf]
