# Supplementary material for: Artificial intelligence framework for multi-pathology risk assessment from retinal fundus images: deep learning approach to 15-disease screening
Source: Front Med (Lausanne). 2026 May 25;13:1778404. doi: 10.3389/fmed.2026.1778404 (PMC13244632; doi:10.3389/fmed.2026.1778404)
Supplement: Supplementary Table S1 — Performance on the patient-level test set (n = 3,981; GroupShuffleSplit partitioning). Three independently trained model variants were evaluated; mean and range (min–max) reported across variants. †Classes with fewer than 100 positive test examples. For comparison, image-level metrics from Table 2: mean AUC = 0.976, mAP = 0.976, macro-F1 = 0.931. [file Table_1.pdf]

Supplementary Table S1. Patient-level performance metrics (GroupShuffleSplit, n=3,981 test images)
Results from three independently trained model variants; range shown as (min–max) across variants.
† Classes with <100 positive test examples; interpret with caution.
Image-level comparisons: mean AUC 0.976, mAP 0.976, macro-F1 0.931 (main text Table 2).

Class	n+ (test)	Mean AUC (min-max)	Mean F1 (min-max)	Mean AP (min-max)	
Atherosclerosis	118	0.887 (0.879–0.895)	0.407 (0.398–0.421)	0.379 (0.369–0.389)	
Cataract	441	0.956 (0.955–0.956)	0.701 (0.696–0.704)	0.777 (0.776–0.778)	
CHPRE	182	0.945 (0.943–0.946)	0.581 (0.576–0.590)	0.641 (0.637–0.650)	
Diabetic Retinopathy	132	0.977 (0.976–0.980)	0.701 (0.695–0.707)	0.805 (0.799–0.808)	
Glaucoma	586	0.995 (0.994–0.995)	0.953 (0.953–0.953)	0.984 (0.983–0.985)	
HIV Retinopathy	55	0.873 (0.862–0.889)	0.454 (0.404–0.500)	0.408 (0.399–0.418)	†
Hypert. Retinopathy	101	0.918 (0.909–0.925)	0.322 (0.314–0.337)	0.306 (0.291–0.326)	
Macular Degeneration	589	0.982 (0.981–0.982)	0.845 (0.840–0.848)	0.914 (0.912–0.915)	
Malformation	20	0.943 (0.937–0.955)	0.456 (0.435–0.488)	0.397 (0.384–0.405)	†
Periph. Ret. Deg.	864	0.954 (0.953–0.954)	0.777 (0.771–0.781)	0.834 (0.832–0.836)	
Pig. Choroidal Neopl.	320	0.921 (0.920–0.923)	0.557 (0.549–0.565)	0.580 (0.567–0.588)	
Retinitis Pigmentosa	111	0.986 (0.984–0.988)	0.815 (0.812–0.818)	0.858 (0.855–0.860)	
Retinoblastoma	19	0.951 (0.950–0.952)	0.915 (0.900–0.923)	0.922 (0.920–0.923)	†
Systemic Lupus	34	0.839 (0.830–0.853)	0.390 (0.351–0.412)	0.354 (0.322–0.384)	†
Vascular Occlusions	142	0.882 (0.871–0.894)	0.465 (0.460–0.470)	0.468 (0.460–0.478)	
**Mean (all classes)**	**—**	**0.934 (0.930–0.939)**	**0.623 (0.619–0.627)**	**0.642 (0.638–0.644)**	
